# Tricuspid Atresia Type 1B With Persistent Left Superior Vena Cava in a Four-Month-Old Child: An Unusual Combination

**DOI:** 10.7759/cureus.14450

**Published:** 2021-04-13

**Authors:** Subrahmanya Murti Velamakanni, Aman Patel, Gajanan Khadkikar, Sanjay C Shah, Tejas Patel

**Affiliations:** 1 Cardiology, Smt. Nathiba Hargovandas Lakhmichand (NHL) Municipal Medical College, Ahmedabad, IND; 2 Interventional Cardiology, Apex Heart Institute, Ahmedabad, IND; 3 Cardiology, Apex Heart Institute, Ahmedabad, IND

**Keywords:** cyanotic congenital heart disease, tricuspid atresia, left superior vena cava (lsvc), coronary sinus (cs)

## Abstract

Tricuspid atresia (TA) is a rare cyanotic congenital heart disease. A persistent left superior vena cava (LSVC) may be associated with TA. The presence of LSVC raises important considerations for eventual repair, in that it may lead to persistent arterial desaturation even after corrective surgery, if associated with an unroofed coronary sinus. Here, we present the case of a four-month-old child who was diagnosed with TA type 1B, LSVC and a dilated coronary sinus by transthoracic echocardiography.

## Introduction

Tricuspid atresia (TA) is a rare cyanotic congenital heart disease (CHD) with an estimated incidence of 79 per million live births [1]. Tricuspid atresia has been classified according to the modified Edward-Burchells classification into three major types based on the relations of the great arteries and degree of pulmonary stenosis [2]. A persistent left superior vena cava (LSVC) may be associated with TA and may lead to persistent arterial desaturation even after corrective surgery if associated with an unroofed coronary sinus [3]. Here, we present the echocardiographic images of a cyanosed four-month-old child who presented with failure to thrive and fatigue on feeding.

This article was previously posted to the Authorea preprint server on March 29, 2021 [4]. The preprint version is not pending full publication elsewhere.

## Case presentation

**Figure 1:**
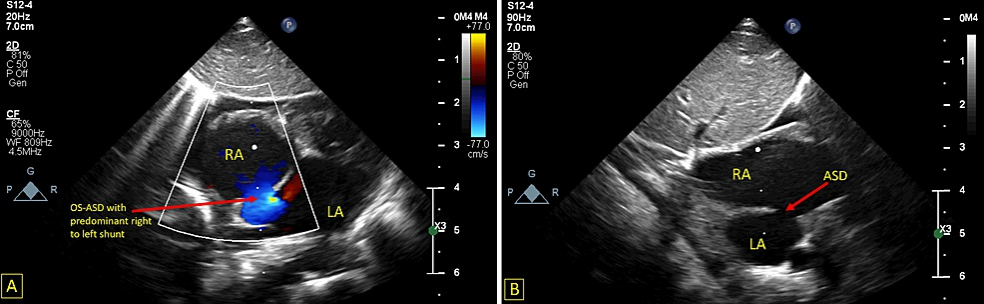
A: Showing a subcostal view with color Doppler demonstrating an ostium secundum atrial septal defect with predominant right to left shunt. B: Subcostal bicaval view showing the atrial septal defect. LA – Left atrium, RA – Right atrium, OS-ASD – Ostium secundum atrial septal defect

**Figure 2:**
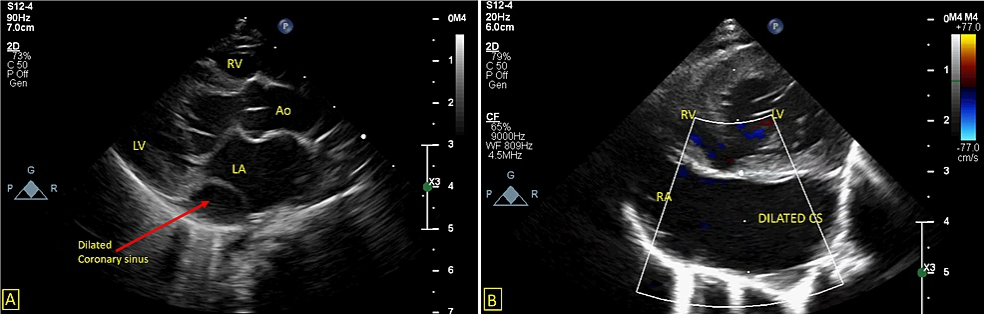
A: Parasternal long axis view showing a dilated coronary sinus. B: Foreshortened apical view showing a dilated coronary sinus. LA – Left atrium, LV – Left ventricle, RA – Right atrium, RV – Right ventricle, Ao – Aorta, CS – Coronary sinus

A four-month-old child was referred for cardiac evaluation with a history of failure to thrive and fatigability on feeding. Peripheral arterial oxygen saturation was 84% and all four limb pulses were normal. On examination, central cyanosis was present along with a grade 4/6 pan-systolic murmur heard at the left lower sternal border. There were no other obvious dysmorphic features. On transthoracic echocardiography, there was situs solitus and levocardia. In the subcostal window, a large ostium secundum atrial septal defect (ASD) was noted with predominant right to left shunting (Figure 1, Videos 1-2). On examination in the parasternal long axis view, a dilated coronary sinus (CS) was noted (Figure 2, Videos 3-4).

**Video 1:** Subcostal view showing an ostium secundum atrial septal defect with predominant right to left shunt

**Video 2:** Subcostal bicaval view showing an ostium secundum atrial septal defect

**Video 3:** Parasternal long axis view showing a dilated coronary sinus

**Video 4:** Foreshortened apical four chamber view showing a dilated coronary sinus

**Figure 3:**
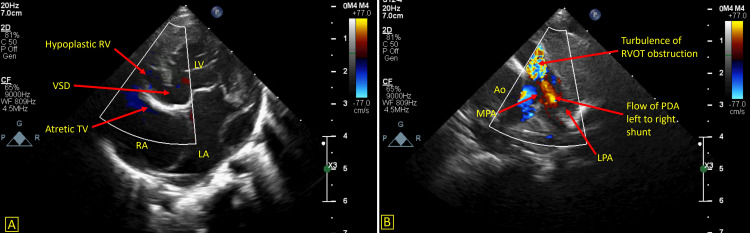
A: Showing an apical four chamber view. B: Color Doppler of the parasternal short axis view showing a patent ductus arteriosus with left to right shunt. LA – Left atrium, LV – Left ventricle, RA – Right atrium, RV – Right ventricle, RVOT – RV outflow tract, Ao – Aorta, TV – Tricuspid valve, VSD – Ventricular septal defect, PDA – Patent ductus arteriosus, MPA – Main pulmonary artery, LPA – Left pulmonary artery.

**Figure 4:**
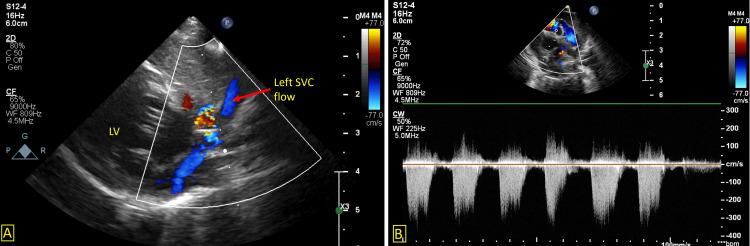
A: Color Doppler of a high parasternal view showing a left superior vena cava. B: Showing a maximum gradient of 50 mmHg on continuous wave Doppler of the right ventricular outflow tract. LV – Left ventricle, SVC – Superior Vena Cava

In the apical four chamber view, an atretic tricuspid valve was seen with no flow across the same. A large inlet ventricular septal defect was also seen with left to right shunting filling a hypoplastic right ventricle, with the left ventricle being dilated (Figure 3A, Video 5). The left ventricular function was normal. The pulmonary valve was morphologically normal, main pulmonary artery was dilated and branch pulmonary arteries were normal. A patent ductus arteriosus was present with a left to right shunt (Figure 3B, Video 6). On modified high parasternal views, LSVC was identified (Figure 4A, Video 7). A right ventricular outflow tract maximum gradient of about 50 mmHg was seen suggesting moderate sub-pulmonary stenosis (Figure 4B). Atrio-ventricular and ventriculo-arterial concordance was preserved. Pulmonary venous return was normal. The right superior and inferior vena cava drained normally. Aortic arch was normal and left sided. Coronary artery origins as traced out on short axis views appeared normal. Thus, a diagnosis of tricuspid atresia type 1B as per the modified Edwards-Burchells classification was made [2]. The child was referred to a specialized pediatric cardiac surgery center for further management and lost to follow up.

**Video 5:** Apical four chamber view showing an atretic tricuspid valve and a ventricular septal defect with left to right shunt

**Video 6:** Parasternal short axis view with color Doppler showing the flow of patent ductus arteriosus with left to right shunt and turbulence in the right ventricle outflow tract suggestive of stenosis

**Video 7:** Modified high parasternal view showing a left superior vena cava

## Discussion

TA has been classified as per the modified Edwards-Burchells scheme [2]. Three types have been described - type I with normally related great vessels, type II with transposed great vessels and type III with a persistent truncus arteriosus. Each type can be further subdivided into three subtypes - subtype A: with pulmonary atresia, subtype B: with pulmonary or sub-pulmonary stenosis and subtype C: without pulmonary or sub-pulmonary stenosis. The present case is that of a type 1B variant.

A persistent LSVC draining into the CS is associated with many congenital heart diseases. In the largest series on the presence of persistent LSVC with congenital heart disease, 88 out of 2663 children (3.3%) had a persistent LSVC [5]. In the same series, there were 53 patients of TA, three of whom had an LSVC (5.6%).

The identification of an LSVC is important for eventual surgical repair. The treatment of choice for patients of TA is the Fontan’s procedure. The original Fontan procedure was performed in a staged manner. The first step was connecting the superior vena cava (SVC) to the right pulmonary artery - known as the Glenn's shunt. This was followed by a staged procedure with four components - connecting the inferior vena cava (IVC) through right atrium (RA) to PA, insertion of a valve in the IVC, closure of the ASD and obliteration of the right ventricle (RV) to PA connection [6]. This has been modified to the present day preferred approach of an extra-cardiac conduit with a fenestration connecting the conduit to the RA. The extra-cardiac conduit eliminates the disadvantage of RA dilation as seen in the atrio-pulmonary Fontan procedure [6]. Thus, the basic concept of the Fontan procedure is the rerouting of the systemic venous drainage directly to the pulmonary arteries, thereby bypassing the hypoplastic right ventricle. In a classical series, unoperated patients of TA with cyanosis had a one-year mortality of 90% [6]. However, in a large series of 225 patients who underwent the Fontan procedure, survival at one month, one year, 10 years and 20 years were 90%, 81%, 70% and 60%, respectively [7].

In most cases, the presence of an LSVC does not change the management plan nor does it create post-operative complications. However, if there is unroofing of the CS with a persistent LSVC, it may cause persistent arterial desaturation despite a shunt surgery, as deoxygenated blood from the LSVC enters the left atrium via the unroofed coronary sinus [8]. Unroofing of the CS may be easily detected by contrast echocardiography. The same finding may be confirmed by cardiac computed tomography as well. Hence, it is important to delineate the presence of a dilated CS and a persistent LSVC before surgery in children with TA or single ventricle physiology.

## Conclusions

TA is a rare cyanotic CHD requiring shunt procedures for palliation. A persistent LSVC draining into a dilated CS may be uncommonly associated with TA. An unroofed CS in the same setting may have important implications for eventual surgery as it may cause persistent arterial desaturation in the post operative period. Therefore, a persistent LSVC must be sought for in all cases of not only TA, but other cyanotic CHDs as well.
